# Graphical Abstract: ChemistryOpen 1/2015

**DOI:** 10.1002/open.201580111

**Published:** 2015-02-18

*Chemistry Open* is a multidisciplinary, gold-road, open-access, international forum for the publication of Full Papers and Communications from all areas of chemistry and related fields. *ChemistryOpen* also publishes the Thesis Treasury containing summaries of Ph.D. theses and links to the full version via our homepage. Based in Europe, *ChemistryOpen* attracts authors and readers from around the world, as open-access publishing becomes more important in all areas of chemistry. *ChemistryOpen* is co-owned by ChemPubSoc Europe and published by Wiley-VCH. Authors can submit their primary research articles and thesis summaries via our homepage by clicking “submit an article”. All contributions considered suitable for publication are subject to peer review, and if accepted, electronically processed and published online ensuring high quality and short publication times.

## COVER PICTURE

**The cover picture shows** coupled and uncoupled states of a [2]rotaxane incorporating stable nitroxide radical units in both the ring and dumbbell components. Interaction between nitroxide radicals could be switched between noncoupled (three-line electron paramagnetic resonance (EPR) spectrum) and coupled (five-line EPR spectrum) upon deprotonation of the rotaxane NH2 + centers that effects a quantitative displacement of a dibenzocrown macroring to a 4,4’-bipyridinium recognition site. The complete base- and acid-induced switching cycle of the EPR pattern was repeated several times without an appreciable loss of signal, highlighting the reversibility of the process. Hence, this molecular machine is capable of switching on/off magnetic interactions by chemically driven reversible mechanical effects. For more details, see the Communication on p. 18 ff.

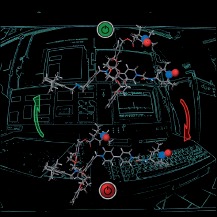



## COVER PROFILE

V. Bleve, C. Sch_fer, P. Franchi, S. Silvi, E. Mezzina,* A. Credi,* M. Lucarini*

Reversible Mechanical Switching of Magnetic Interactions in a Molecular Shuttle

*“This molecular machine is capable of switching on/off magnetic interactions by chemically driven reversible mechanical effects. A system of this kind represents a first step towards a new generation of nanoscale magnetic switches that may be of interest for information and communication technology (ICT) applications.”* More about the story behind the cover research can be found on p. 2.

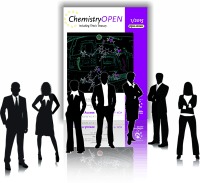



## News

Spotlights on our sister journals

10 – 13

## EDITORIAL

N. Ortfflzar*

14 – 17

**Open-Access Chemistry with Impact**

First on the scene: At launch, *ChemistryOpen* was the first societyowned general chemistry journal to publish open access articles exclusively. Now with an impressive first impact factor of 2.938, read how the journal has developed and grown over the first three years into a high-quality journal with impact.

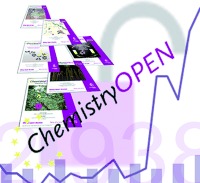



## COMMUNICATIONS

V. Bleve, C. Schäfer, P. Franchi, S. Silvi, E. Mezzina,* A. Credi,* M. Lucarini*

18 – 21

Reversible Mechanical Switching of

Magnetic Interactions in a Molecular Shuttle

**It’s shuttle time!** We developed an acid–base switchable molecular shuttle based on a [2]rotaxane, incorporating stable radical units in both the ring and dumbbell components. The interaction between the radicals can be reversibly switched from uncoupled (upon acid addition) to coupled (upon base addition). Hence, this molecular machine is capable of switching on/off magnetic interactions by chemically driven reversible mechanical effects.

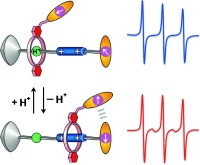



S. J. Cloake, H. S. Toh, P. T. Lee, C. Salter, C. Johnston, R. G. Compton*

22 – 26

Anodic Stripping Voltammetry of Silver Nanoparticles: Aggregation Leads to Incomplete Stripping

**Things to consider when stripping:**Aggregation causes incomplete stripping of dopamine-capped silver nanoparticles. Two possible mechanisms of (A) ‘partial oxidation’ and (B) ‘inactivation’ of the nanoparticles are proposed to account for incomplete stripping. Aggregation effects must be considered when anodic stripping voltammetry is used for nanoparticle detection and quantification.

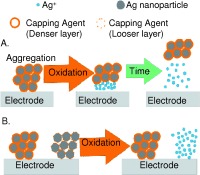



M. Nguyen, L. Rechignat, A. Robert,* B. Meunier*

27 – 31

The Necessity of Having a Tetradentate Ligand to Extract Copper(II) Ions from Amyloids

**Clearing out the Cu in AD:** Bis(8-aminoquinoline) ligands are drug candidates able to extract CuII from copperloaded amyloid peptides (Aβ) in order to restore copper homeostasis in the brain of patients. Contrarily, in the presence of an 8-hydroxyquinoline such as clioquinol, the copper ions remain sequestrated within an Aβ–Cu–clioquinol ternary complex.

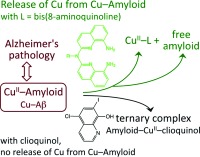



C. Wei, C. Cheng, J. Zhao, Z. Wang, H. Wu, K. Gu, W. Du, H. Pang*

32 – 38

Mesoporous ZnS–NiS Nanocomposites for Nonenzymatic Electrochemical Glucose Sensors

## FULL PAPERS

Sensing something sweet: Mesoporous ZnS–NiS composites, prepared via ionexchange reactions using ZnS as the precursor, exhibited a high specific surface area and a rational composition of the two constituents. Electrochemical sensors based on these composites exhibited high selectivity, a stable signal, and a low detection limit of 0.125 μm for glucose, providing an alternative to enzyme-based glucose sensors.

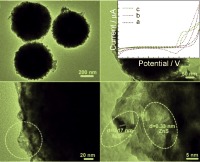



T. Rosholm, P. M. P. Gois, R. Franzen, N. R. Candeias*

39 – 46

Glycerol as an Efficient Medium for the Petasis Borono–Mannich Reaction

**Enjoy a different solvent!** Glycerol is explored as an effective medium for the multicomponent Petasis borono–Mannich (PBM) reaction with aromatic aldehydes. Alkylaminophenols, 2-substituted pyridines, and 2*H*-chromenes were prepared in reasonable to good yields. Interestingly, crude glycerol, a byproduct of the biodiesel industry, was used for the PBM reaction successfully. A mechanistic study of the reaction based on density functional theory was also performed.

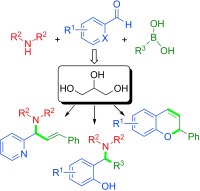



C. Fontana, A. Weintraub, G. Widmalm*

47 – 55

Structural Elucidation of the -Antigen Polysaccharide rom *Escherichia coli* O181

**Sugars from Shiga!** The native O-antigen polysaccharide of Escherichia coli O181 and the oligosaccharide material obtained by its dephosphorylation with 48% hydrofluoric acid have been characterized by NMR spectroscopy. Both polysaccharides (native and O-deacetylated) and the oligosaccharide display an unusual conformational behavior that causes some key resonances of the lcNAc residue to broaden at temperatures elow 85ºC.

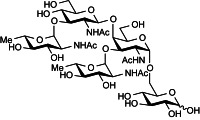



N. B. Vinh, S. M. Devine, L. Munoz, R. M. Ryan, B. H. Wang, H. Krum, D. K. Chalmers, J. S. Simpson, P. J. Scammells*

56 – 64

Design, Synthesis, and Biological Evaluation of Tetra-Substituted Thiophenes as Inhibitors of p38α MAPK

**Dock and design!** A library of substituted thiophenes bearing the vicinal 4-fluorophenyl/ 4-pyridyl rings was designed using computational docking as a visualization tool. Compounds were synthesized and evaluated as p38α mitogen-activated protein kinase (MAPK) inhibitors in a fluorescence polarisation binding assay. Several compounds exhibited low micromolar affinity to the active form of p38α MAPK. Selected analogs were also shown to suppress neonatal rat fibroblast collagen synthesis.

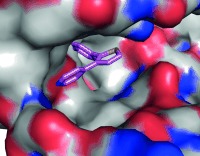



C. Schjoeth-Eskesen, P. R. Hansen, A. Kjaer, N. Gillings*

65 – 71

Efficient Regioselective Ring Opening of Activated Aziridine-2-Carboxylates with [^18^F]Fluoride

Strain relief with ^18^F: Reaction of activated aziridine-2-carboxylate with nucleophilic [^18^F]fluoride shows complete regioselectivity at the most substituted carbon atom. Following deprotection, this enabled the efficient radiosynthesis of α-[^18^F]fluoro-β-alanine with the use of tert-butyloxycarbonyl (Boc) or carboxybenzyl (Cbz) as activating groups.

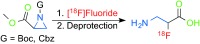



## THESIS TREASURY

H. M. Abdelaal*

72 – 75

Facile Hydrothermal Fabrication of Nano-Oxide Hollow Spheres using Monosaccharides as Sacrificial Templates

**Sugar makes me hollow inside!** This thesis presents a facile, environmentally benign, economically sustainable, and additive-free synthetic protocol for the fabrication of hollow oxides, which could ultimately enable the fine-tuning of hollow inorganic materials. The synthesis was achieved via a facile one-pot hydrothermal strategy with the use of simple sugars (glucose or fructose) as sacrificial templates.

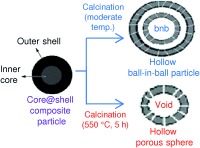



## SERVICE

* Author to whom correspondence should be addressed.

Supporting information is available on the WWW (see article for access details).

This is an open-access article, published under the terms and conditions of a Creative Commons License, as stated in the final article.

A video clip is available as Supporting Information on the WWW (see article for access details).

Contributions labeled with this symbol have been judged as “Very Important Papers” by the referees.

All the Tables of Contents may be found on the WWW under: http://www.chemistryopen.org

